# Genome sequence of the novel Cystobasidiomycetes fungal isolate EMM_F5

**DOI:** 10.1128/mra.00718-25

**Published:** 2025-10-29

**Authors:** Sachida Pokhrel, Zachary A. Noel

**Affiliations:** 1 Department of Entomology and Plant Pathology, Auburn University 1383, Auburn, Alabama, USA; University of California Riverside, Riverside, California, USA

**Keywords:** fungi, genome, Cystobasidiomycetes

## Abstract

The whole genome of fungal isolate EMM_F5, isolated from the phyllosphere of *Magnolia grandiflora,* was sequenced, assembled, and annotated. Preliminary phylogenomic analysis places EMM_F5 in the Cystobasidiomycetes class within Basidiomycota fungi, with multigene phylogenies placing it in Microsporomycetaceae family.

## ANNOUNCEMENT

Yeasts are known colonizers of the phyllosphere habitat (1, 2), though many remain unclassified (3).

**Fig 1:**
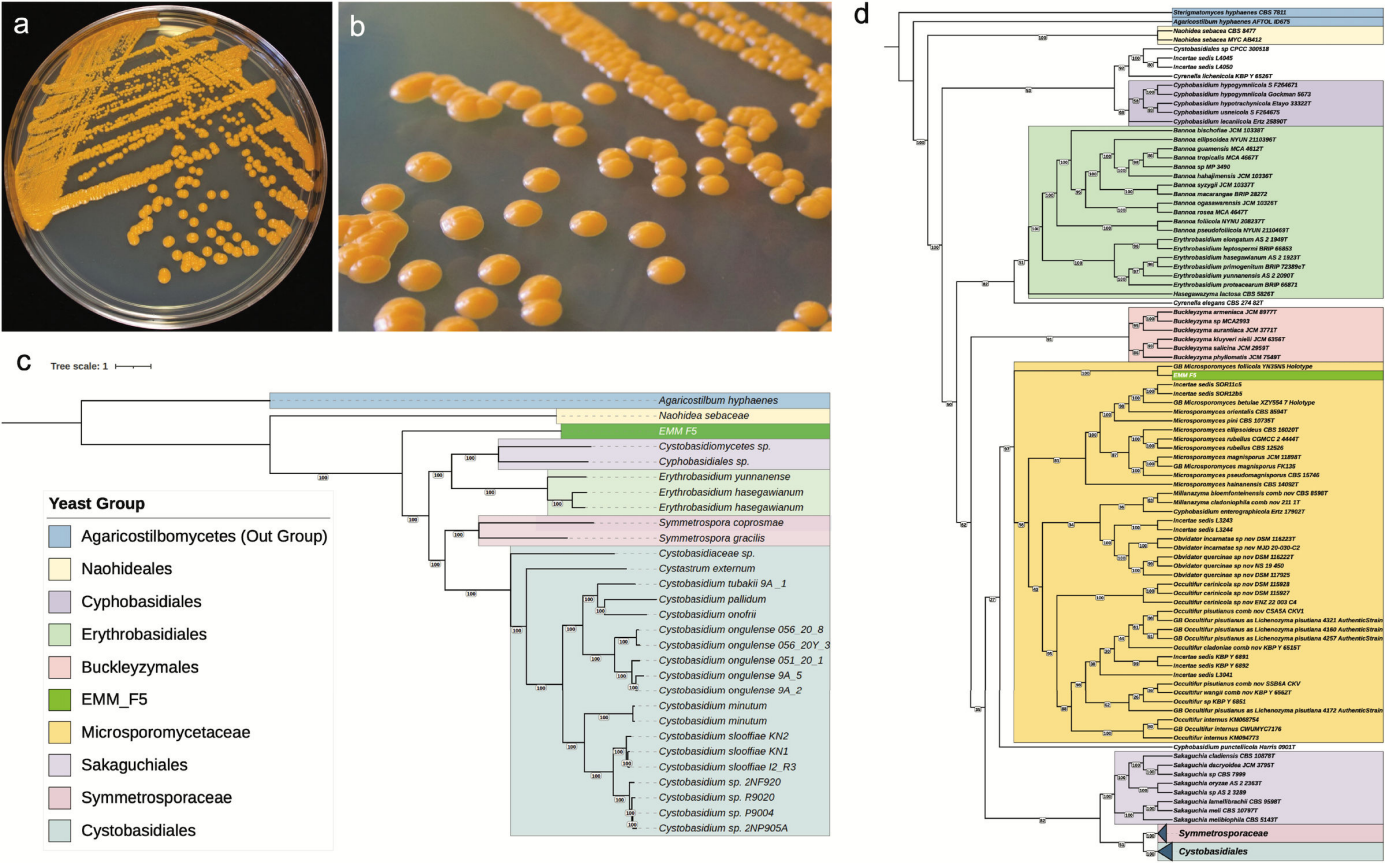
(**a**) EMM_F5 on malt extract agar amended with yeast extract. (**b**) Close-up colonies of EMM_F5. (**c**) Maximum likelihood tree generated using IQ-TREE (4) through Phyling v2.2.0 based on 1708 BUSCO orthologs aligned against basidiomycota Hidden Markov Model (HMM) marker set from the predicted protein sequences (5) and visualized using iTOL (6). Repeat masking of the NCBI genomes and EMM_F5 was conducted using tantan (7) via the “mask” function in funannotate v1.8.13 (8) before protein prediction. *Agaricostilbum hyphaenes*, a taxon belonging to Agaricostilbomycetes, was used as an outgroup. The number at the branches designates the percent bootstrap value out of 1,000 iterations. (**d**) Maximum likelihood tree generated using IQ-TREE (4) using seven genetic markers; ITS, SSU, LSU, RPB1, RPB2, TEF1a, and CYTb partitioned to use their own models for sequence evolution and visualized using iTOL (6). Agaricostilbomycetes was used as an outgroup for the phylogenetic placement of EMM_F5. The number at the branches designates the percent bootstrap value out of 1,000 iterations.

We isolated a yeast, EMM_F5, from the phyllosphere of *Magnolia grandiflora* in the Donald E. Davis Arboretum at Auburn University (32.5959° N, 85.4828° W) on 21 January 2021. Leaf strips approximately 4 cm by 10 cm long were cut with flame-sterilized scissors into a sterile 50 mL tube. Strips were vortexed in 1× phosphate-buffered saline for 1 min, 100 μL was spread onto Petri dishes containing Difco malt extract agar (BD Biosciences, NJ, USA) amended with 1 g yeast extract (ME+) (MP Biomedicals, OH, USA), supplemented with rifampicin (0.01 mg/mL) and chloramphenicol (0.1 mg/mL). Individual colonies were subcultured onto new ME+ agar to represent an isolate. EMM_F5 had yellow-orange colonies with butyrous texture (Fig. 1a and b). Dalmau’s technique on cornmeal agar confirmed yeast-like morphology. The ITS region (PV764649) showed 82.24% sequence identity (100% query coverage) with *Erythrobasidium hasegawianum* CBS 10217 (EU002885.1).

For genomic DNA isolation, a single colony of EMM_F5, grown on ME+ agar, was inoculated into 5 mL of ME+ broth and grown for 3 days at 28°C. The cells were collected via centrifugation, and the pellet was processed using the E.Z.N.A. Plant DNA DS Kit (Norcross, GA). A library was prepared using the Illumina DNA Prep kit and IDT 10 bp UDI indices and sequenced on the Illumina NovaSeq 6000, generating 2 × 151 bp paired-end reads. Quality filtering and adapter removal were done with Trimmomatic v0.39 (9). *De novo* genome assembly used SPAdes v3.15.5 (10) followed by decontamination with FCS-GX v0.5.0 (11). Assembly quality was evaluated using QUAST v5.2.0 (12), and completeness was assessed using BUSCO v5.4.3 (13) using the basidiomycota_odb10 data set.

Sequencing yielded a total of 2,630,670,570 bp with a Q-score >30 for 93.53% of reads. The final assembled genome was 18,587,251 bp in length (coverage = 142×), comprising 253 contigs ≥ 500 bp, with a GC content of 52% and an N50 of 529,534 bp. Funannotate v1.8.13 (8) was used for the prediction of protein sequences from the genome using the “predict” function and annotation of protein sequences using output data from eggnog/2.1.7 (14) and InterProScan/5.66 (15) using the “annotate” function. Coding sequence (CDS) regions accounted for 10,719,149 bp, and 6,580 protein-coding genes were predicted using Funannotate v1.8.13. Gene prediction using BUSCO training identified 1,524 complete genes (86.4%).

A phylogenomic analysis with 27 Cystobasidiomycetes genomes and one Agaricostilbomycetes outgroup (from JGI MycoCosm and NCBI) placed EMM_F5 within Cystobasidiomycetes, lacking a sister taxon (Fig. 1c). As no genome data exist for Microsporomycetaceae and Sakaguchiales, we collated sequences from (16) and *Microsporomyces follicola* YN35N5T (17), comparing seven EMM_F5 genes (ITS, SSU, LSU, TEF1α, RPB1, RPB2, Cytb) using IQ-TREE (Fig. 1d). EMM_F5 clustered with *Microsporomyces follicola* YN35N5T.

We hypothesize that EMM_F5 belongs to Microsporomycetaceae, potentially representing the first genomic resource for this family and highlighting a gap in fungal genomics.

## Data Availability

The raw forward and reverse sequences for EMM_F5 are available on the Sequence Read Archive accession number SRR30574311 under BioProject PRJNA1153973. This Whole Genome Shotgun project has been deposited at DDBJ/ENA/GenBank under the accession JBPGPT000000000. The ITS sequence accession number for EMM_F5 is PV764649. Default parameters were used for all software except where otherwise noted. The strain can be obtained by contacting the Noel lab at Auburn University while in the process of being deposited to a culture collection.
